# The design of behavioural interventions labelled as patient‐mediated: A scoping review

**DOI:** 10.1111/hex.12653

**Published:** 2017-11-09

**Authors:** Jeremy Y. Ng, Anna R. Gagliardi

**Affiliations:** ^1^ Toronto General Hospital Research Institute University Health Network Toronto ON Canada

**Keywords:** behaviour change, intervention, patient‐mediated intervention, quality of care, scoping review

## Abstract

**Objective:**

Patient‐mediated interventions (PMIs) directed at patients and/or physicians improve patient or provider behaviour and patient outcomes. However, what constitutes a PMI is not clear. This study described interventions explicitly labelled as “patient‐mediated” in primary research.

**Methods:**

MEDLINE, EMBASE, Allied and Complementary Medicine, PsychINFO, HealthSTAR, Social Work Abstracts, CINAHL and Cochrane Library were searched from inception on 1 January 2017 for English language studies that developed or evaluated behavioural interventions referred to as “patient‐mediated” or “patient mediated” in the full text. Screening and data extraction were independently duplicated. Data were extracted and summarized on study and intervention characteristics. Interventions were categorized as 1 of 4 PMI pathways.

**Results:**

Eight studies (4 randomized controlled trials, 1 observational study and 3 qualitative studies) were included. No studies explicitly defined PMI, and few PMIs were described in terms of content and format. Although 3 studies employed physician interventions, only patient interventions were considered PMIs. One study achieved positive improvement in patient behaviour.

**Conclusions:**

Research is needed to generate consensus on the PMI concept, employ theory when designing or evaluating PMIs, establish the effectiveness of different types of PMIs, and understand when and how to employ PMIs alone or combined with other interventions.

## 1 BACKGROUND

Engaging patients, which includes consumers, the public, family members or care partners, in their own care and in planning or evaluating health service delivery improves patient outcomes and lowers costs.^1^ Hence, patients are key health‐care stakeholders and central to health‐care quality improvement. Yet implementation research, the study of behavioural determinants and interventions that optimize health‐care quality, has largely focused on health‐care professionals.^2^, ^3^ The paucity of research on “patient‐mediated interventions” (PMIs) is notable—interventions targeting patients can have moderate to large effects on health‐care delivery and associated outcomes,^4^ and some research suggests that interventions aimed at both patients and providers may be more effective than targeting one group alone.^5^

A recent editorial on topics of relevance to the field of implementation science highlighted a lack of clarity on what constitutes a PMI.^2^ The Cochrane Effective Practice and Organisation of Care (EPOC) Review Group categorizes PMI as an implementation strategy targeted at health‐care professionals and defines a PMI as “the use of patients, for example, by providing patient outcomes, to change professional practice.”^6^ The definition does not specify if patients themselves report outcomes to health‐care professionals or data about patient outcomes are provided by others to health‐care professionals, a mechanism that also underlies other EPOC implementation strategies targeting health‐care professionals including audit and feedback, clinical incident reporting, educational outreach, public release of performance data and routine patient‐reported outcome measures. Researchers have more expansively defined PMIs as “any intervention aimed at changing the performance of health‐care professionals through interaction with patients, or information provided by or to patients.”^5^ In this definition, patients either report outcomes to health‐care professionals or first receive information that presumably influences their interaction with and/or the behaviour of health‐care professionals. The Expert Recommendations for Implementing Change (ERIC) checklist of 73 implementation strategies does not include a category labelled as PMI, but does include interventions in which patients receive information, which are labelled as educational meetings, involve patients and family members, prepare patients to be active participants and mass media.^7^ Checklists of behavioural interventions (EPOC, ERIC) differ in whether and how they recognize and define PMIs,^6^, ^7^ leaving researchers who wish to evaluate PMIs to devise their own broad definitions,^5^ and offering no clear guidance to health‐care professionals responsible for quality improvement for what constitutes a PMI.

While patient education was acknowledged as a strategy for improving health‐care delivery and outcomes at least as far back as 1977,^8^ the concept of PMIs may have first emerged in 1992 when Davis et al published a systematic review of 50 randomized controlled trials evaluating the effectiveness of continuing medical education interventions. The review found that providing patients with information and education and providing physicians with patient information were both effective techniques in altering physician performance and patient outcomes. Subsequently, the term “patient‐mediated” was used in 2 systematic reviews that both assessed the effectiveness of interventions for changing physician behaviour. A systematic review by Oxman et al of 102 trials published from 1970 to 1993 explicitly defined PMI as “any intervention aimed at changing the performance of health‐care providers for which information was sought from or given directly to patients by others (ie, direct mailing to patients, patient counselling delivered by others or clinical information collected directly from patients and given to the provider).”^10^ The Oxman et al review found that patient education and patient educational material significantly improved physician performance either alone or combined with physician education. A systematic review by Davis et al of 99 trials published from 1975 to 1994 also included PMIs, although they were not explicitly defined and found that physician‐targeted (ie. providing physicians with data about patient test results or health status, physician education) and patient‐targeted (ie, patient education, patient reminders) PMIs achieved improvements in physician behaviour, patient behaviour and/or patient outcomes.^11^ Even the researchers who first employed the term were inconsistent and vague in how they defined PMIs.

**Figure 1:**
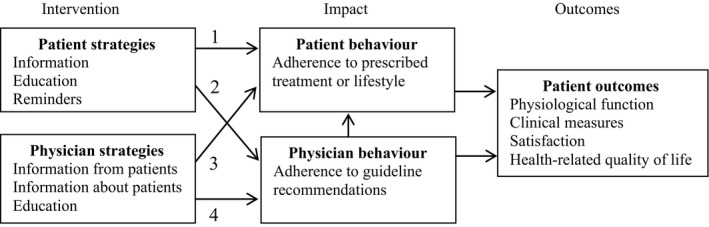
Conceptual framework of patient‐mediated intervention pathways

Inconsistent description, characterization or operationalization of PMIs may limit scientific advancement. Given patient centrality to health care and the demonstrated potential for PMIs to improve health‐care quality,^4^, ^5^ further research is needed to conceptualize PMIs, establish the effectiveness of different types of PMIs and understand when and how to employ them. The EPOC definition of PMI positions the patient (or information about the patient) as the mediator or influencer of health‐care professional behaviour only.^6^ Our review of the original conceptualization of PMI by Oxman^10^ and Davis,^9^, ^11^ and then others,^5^ reveals 4 different pathways that may all mediate or influence a variety of patient outcomes: patient strategies (ie, information, education, reminders) that directly influence patient behaviour or indirectly influence health‐care professional behaviour, and health‐care professional strategies (ie, patients directly report outcomes to physicians, others report patient outcomes to physicians, physician education) that directly influence health‐care professional behaviour or indirectly influence patient behaviour. A depiction of the 4 PMI pathways is shown in Figure 1. This preliminary conceptual framework would benefit from further elaboration of the 4 pathways and associated outcomes, and how they are unique from each other.

To date, PMIs have been inconsistently defined, characterized and operationalized. It is unclear what PMIs are, how they work and to whom they should be directed. Previous systematic reviews that employed the term PMI were published in 1995 and included primary studies published from 1970 to 1994. No subsequent research explicitly sought to conceptualize PMIs by describing interventions labelled as PMIs. The purpose of this research was to conduct a scoping review of studies that developed or evaluated interventions that were explicitly labelled as PMIs, which aimed to improve patient or provider behaviour or patient outcomes, and describe the nature of that research and the characteristics of interventions considered to be PMIs. Such knowledge is needed to identify how the PMI concept has evolved, if all PMI pathways have been investigated, and issues warranting further research. This would more fully establish and distinguish effective PMIs, leading to more consistent use and understanding of this concept, thereby strengthening the underlying science. Ultimately, with this knowledge, those responsible for quality improvement could better select and employ PMIs to enhance health‐care delivery and associated outcomes.

## 2 METHODS

### 2.1 Approach

The overall aim of this research was to describe the characteristics of primary studies that investigated interventions explicitly labelled as PMIs rather than evaluating outcomes or determinants of those outcomes as a means of assessing the effectiveness of PMIs as would a traditional systematic review. A protocol for a Cochrane systematic review has been registered to investigate whether and how PMIs improve professional practice.^12^ Interventions of interest outlined in the protocol include patient information, education, decision aids and membership on committees; information collected from patients given to health‐care professionals; and education of health‐care professionals by patients.^12^ These interventions correspond to pathways 2 and 4 in Figure 1, which focus solely on changing physician behaviour as a means of influencing patient outcomes. Instead, a scoping review was conducted to describe the nature of research on PMIs according to the broader conceptualization of PMIs informed by prior research^9^, ^10^, ^11^ and depicted in Figure 1 and to identify issues not addressed that warrant further research. The scoping review included searching, screening, data extraction and data analysis.^13^ Preliminary exploratory searching, typically the first step, was not needed because the review sought only studies that explicitly employed the term “patient‐mediated” or “patient mediated.”^13^ While not typical of a scoping review, data on outcomes and determinants (enablers, barriers) of those outcomes were extracted, if available, to thoroughly convey the characteristics of research on interventions explicitly labelled as PMIs. The Preferred Reporting Items for Systematic Reviews and Meta‐Analyses (PRISMA) criteria guided reporting of the methods and findings.^14^ Data were publicly available so institutional review board approval was not necessary. A protocol for this review was not registered.

### 2.2 Searching

Several databases were searched on 1 January 2017 from their inception for English language studies that described PMIs: MEDLINE (1946+), EMBASE (1947+), Allied and Complementary Medicine (1985+), PsychINFO (1967+), HealthSTAR (1966+), Social Work Abstracts (1968+), CINAHL (1997+) and Cochrane Library (1946+). To identify studies that explicitly referred to PMIs, the search strategy searched for the terms “patient mediated” or “patient‐mediated” anywhere in the full text of articles.

### 2.3 Eligibility criteria

The eligibility criteria used to screen search results were based on the PICO (population, intervention, comparisons, outcomes) framework. The population of interest included adult patients with any disease or condition, or practicing physicians of any specialty in any setting of care. The intervention of interest was any intervention delivered at any time point or in any setting by health‐care professionals, researchers or others targeted at patients or physicians that was explicitly labelled as a PMI or strategy. With respect to research design, studies could be quantitative (ie, randomized or pragmatic controlled trials, time series, cohort studies—retrospective, prospective, before‐after, multicentre) or qualitative (ie, interviews, focus groups, qualitative case studies) or mixed methods in nature that developed or evaluated a PMI. Systematic reviews were not eligible, but references were screened for eligible primary studies. Studies that included comparisons may have evaluated single or multiple PMIs alone, or compared with no intervention described as usual care or control, a different single or multiple PMI or another type of behavioural intervention. Outcomes of interest were any reported impact of PMIs including beneficial or harmful patient or physician experience, behaviour or outcome. For patients, this included, but was not limited to, adherence to prescribed behaviour or care, physiological function, overall well‐being, return to daily living, pain, social or psychological factors, or adoption of new activities or behaviours, measured clinically, or with instruments, questionnaires or interviews. For physicians, this included, but was not limited to, knowledge or behaviour, measured as adherence to guideline recommendations or indicators.

As search results were reviewed, selection criteria were expanded to specify studies that were not eligible. Studies were excluded if they used the term “patient‐mediated” to refer to patient factors that were facilitators or barriers of seeking care or of compliance with recommended care or to refer to patient outcomes, focused on informational interventions that patients themselves offer or seek (ie, social media), interventions delivered in non‐health–care contexts (ie, business, school), clinical interventions (tests, procedures, treatment), system level interventions (ie, user fees, policies), patient or health‐care professional attitudes in general about patient engagement in their own care or in designing or evaluating health‐care services, or if they were publications in the form of guidelines, conference proceedings or abstracts, protocols, letters, editorials or commentaries.

### 2.4 Screening

Titles and abstracts were screened independently by JYN and ARG. All items selected by at least one reviewer were retrieved for further assessment. If more than one publication described a single study and each presented the same data, the most recent was included.

### 2.5 Data extraction

A data extraction form was developed to collect information on author, year, country, health‐care topic, research design, intervention design according to the Workgroup for Intervention Development and Evaluation Research (WIDER) criteria (theory, single or multifaceted, participants, personnel, content, delivery, timing),^15^ and reported intervention impact including outcomes, facilitators, barriers and harms. JYN and ARG pilot‐tested the form on 3 articles through 2 iterations of data extraction and discussion until data extracted were consistent. Data from all eligible studies were extracted by JYN and independently assessed by ARG.

### 2.6 Data analysis

Summary statistics were used to describe the number of studies by year published, country, health‐care topic and research design, and the number that used theory, single or multifaceted interventions and explicitly defined PMI. Given the sparse and succinct nature of extracted data on PMIs, qualitative content analysis was not possible. Instead, details about intervention characteristics were summarized using text according to where and how PMI was described in the article, target of the intervention (patient, physician), the type of intervention developed or evaluated and intervention impact. Based on a summary of intervention target, intervention and impact, the PMI pathway of interventions developed or evaluated in each study was categorized as 1 of 4 PMI pathways depicted in Figure 1. Given that only half of the included studies reported sparse data on enablers or barriers, detailed analysis using a framework specifying determinants of the adoption of innovations was not performed. The quality of individual studies was not assessed because that is not customary for a scoping review.^13^

## 3 RESULTS

### 3.1 Search results

**Figure 2:**
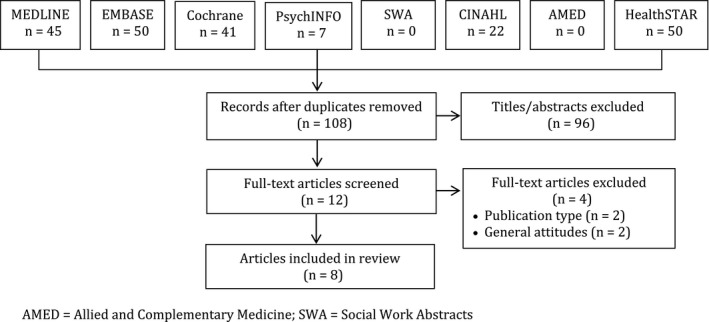
PRISMA diagram

Searches retrieved 215 items in total of which 108 were unique. Title and abstract screening eliminated 96 items. Of the 12 full‐text articles retrieved, 4 were excluded (2 due to publication type and 2 assessed general attitudes about patient engagement). A total of 8 studies were included in the review (Figure 2).

### 3.2 Study characteristics

Table 1 summarizes data extracted from included studies.^16^, ^17^, ^18^, ^19^, ^20^, ^21^, ^22^, ^23^ Studies were published from 2002 to 2015 in Australia (2), Canada (1), Denmark (1), the Netherlands (1), Norway (1) and the United Kingdom (2). Health‐care issues included quality of care for chronic obstructive pulmonary disease (2), community re‐engagement following stroke (1), quality of fertility care (1), secondary prevention of coronary heart disease (1), monitoring of adverse effects of arrhythmia treatment with amiodarone (1), reducing frequent attenders of after‐hours primary care services (1), and rapid return to work after leave for low back pain (1). Research designs included randomized controlled trials (3), randomized controlled trial plus qualitative interviews (1), comparative cohort study (1), qualitative interviews (1) and qualitative focus groups (2). None of the interventions were informed by explicitly named theory. Four studies (50.0%) assessed multifaceted interventions.

**Table 1:** Study and intervention characteristics

Study Health‐care issue Research design PMI	Intervention	Results
McKellar^16^ 2015CanadaCommunity re‐engagement following a strokeRandomized controlled trial plus qualitative interviews	TheoryNRMultifacetedYesContentIntervention group received information on how to communicate with health‐care professionals; the Community Re‐engagement Cue to Action Trigger Tool (CRCATT) covering health, where the patient lives, getting around, social support, life roles, caregiver support, communication and money matters; and a visit to orient the patient to CRCATT and a second visit to reinforce use of the CRCATT. The control group received only the information on how to communicate with health‐care professionals.DeliveryBooklet (communication with health‐care professionals) and question prompt list (CRCATT).TimingVisits were 20 min at 2 and 4 wk following admission to inpatient or outpatient rehabilitation.ParticipantsPatients at 3 rehabilitation hospitals in one city with a primary diagnosis of stroke.PersonnelResearch coordinator conducted follow‐up visits	OutcomesThe main outcome measure was self‐reported participation in valued activities using the validated Reintegration to Normal Living Index (RNLI). Outcomes were reported for 31 intervention and 26 control patients. There was no significant difference between groups in RNLI scores (*t* test 0.163, *P* = .87; *F* = 2.176, *P* > .072).Facilitators/BarriersQualitative interviews were conducted with 16 intervention and 19 control patients. Patients said that a range of informal (caregivers, friends, family) and formal (programmes) sources of support were important to overall recovery. CRCATT was considered useful but patients also valued interactions with health‐care professionals to access information and answers about their prognosis and recovery needs. Intervention patients were more likely to say they initiated question‐asking and discussions and had a positive interaction with health‐care professionals.HarmsNR
Huppelschoten^17^2015The NetherlandsFertility careRandomized control trial	TheoryNRMultifacetedYesContentAudit and feedback report consisted of data on patient‐centredness collected by questionnaire from a random selection of fertility clinic's own patients. Educational outreach focused on discussion of audit and feedback report to jointly define improvement goals and concrete actions to achieve those goals; and health‐care teams were informed about different PMIs they could implement to enhance communication with patients (ie, organizing focus groups); online community for health‐care professionals and patients to exchange ideas about improvement; newsletter provided progress reportDeliveryAudit and feedback; educational outreach visit; patient‐mediated intervention (PMI); online community discussion board; newsletterTimingEducational outreach visit conducted 2 wk after clinics received audit and feedback report. A researcher called health‐care teams every 2 mo to monitor progress. Clinics received a newsletter every 2 mo.ParticipantsHealth‐care teams at 16 of 32 fertility clinics that were randomized to intervention. Health‐care teams were responsible for choosing and implementing PMIs. Researchers performed educational outreach visits. One female patient and her partner recruited by the Dutch patient association and trained prior to study, and a quality officer also participated in educational outreach visits.PersonnelThe fertility teams and the authors of the manuscript	OutcomesMain outcome measure was patient‐reported patient‐centredness based on the validated Patient‐Centredness Questionnaire‐Infertility (PCQ‐Infertility). Outcomes reported for 367 intervention and 329 control patients at baseline, and 377 intervention and 353 control patients after the intervention. At baseline, there was no significant difference in PCQ‐Infertility between intervention and control patients. After intervention, there was no significant difference in PCQ‐Infertility between intervention and control patients (*B* = 0.06, 95% CI −0.04 to 0.15, *P* = .25). In subscale analysis, only “continuity of care” was significantly higher in the intervention compared with control group (*B* = 0.20, 95% CI 0.0‐0.4, *P* < .05)Facilitators/BarriersNRHarmsNR
Harris^18^2010AustraliaChronic obstructive pulmonary disease (COPD)Qualitative interviews	TheoryNRMultifacetedNoContent22 summaries of evidence on treatments for COPD; each summary accompanied by a suggested question to discuss with their doctor to prompt consideration/use of the evidence; written in lay language; questions were written with health‐care professionals; instructions to read the most relevant sections first, take the manual to consultations and discuss topics with their general practitionerDeliveryPatient manual of 80 pages, small page size, large print, question‐and‐answer format, illustrationsTimingSingle meeting with patients to provide manual; follow‐up interview took place between 3 and 12 mo after receiving manualParticipants16 patients (8 were female) interviewed of 125 who received COPD manual in previous trial^19^ who ranged in age from 45 to 90 yPersonnelStudy researchers	OutcomesNRFacilitators/BarriersParticipants varied in level of interest in the manual, views about relevance, usefulness and benefits of the content; and views about the usefulness of the design of the manual. No participants asked questions offered in the manual; few said they asked other questions. They said they could raise questions when they wanted to, although were aware of consultation time limits. Participants did not see advantages in asking questions suggested in the manual. The manual was seen as containing medically oriented information that was relevant to physicians; they were not viewed as topics that the patient would raise in consultations.HarmsNR
Harris^19^2006AustraliaChronic obstructive pulmonary disease (COPD)Comparative cohort study	TheoryNRMultifacetedNoContent22 summaries of evidence on treatments for COPD; each summary accompanied by a suggested question to discuss with their doctor to prompt consideration/use of the evidenceDeliveryPatient manual of 80 pagesTimingSingle meeting with patients to provide manual; follow‐up questionnaire administered by interview in person or by telephone at 3 moParticipantsPatients attending 3 hospitals in South Australia with moderate/severe COPDPersonnelResearcher provided patients with manual	OutcomesMain outcome measures were self‐reported influenza vaccination within previous 15 mo and bone density testing within previous 42 mo based on researcher administered questionnaire. Outcomes reported after the intervention only for 115 intervention and 117 control patients. There were no statistically significant differences between intervention and control for influenza vaccination or bone density testing. In subanalyses, intervention patients from socio‐economically disadvantaged areas had significantly higher rates of bone density testing (*P* = .035).Facilitators/BarriersNRHarmsNR
Murie^20^2006United KingdomSecondary prevention of coronary heart disease after myocardial infarctionQualitative focus group	TheoryNRMultifacetedNoContentInformation about congestive heart failure, treatment options and how to choose from among them and information on how to make lifestyle changesDeliveryBookletTimingOn single occasion patients reviewed samples of information (ie, leaflets, manuals), identified what they viewed as the best/most appropriate information, then described the ideal content and format of a patient bookletParticipants6 post‐myocardial infarction patients (2 was female) from a single practice ranging in age from 45 to 68PersonnelStudy researcher	OutcomesNRFacilitators/BarriersPatients varied in their understanding of the risks associated with cardiac surgery. Information was essential but sometimes introduced to early in hospital. Patients valued personalized targets and treatment plans. Shared decision making had been experienced for lifestyle changes but was not considered appropriate for cardiac surgery. Patients desired information on self‐management with a positive tone and unambiguous guidance for both the inpatient stage and early post‐discharge stage. Important features included visual appeal and small format that could be carried away.HarmsNR
Murie^21^2005United KingdomMonitoring of adverse effects of arrhythmia treatment with amiodaroneQualitative focus group	TheoryNRMultifacetedNoContentInformation about risk of adverse effects associated with amiodarone in congestive heart failure or after myocardial infarctionDeliveryBookletTimingShown to patients on a single occasion to evaluate content/formatParticipants6 patients (3 were female) from a single practice evaluated and discussed the booklet; mean age was 69 y (range 57‐80)PersonnelStudy researchers	OutcomesNRFacilitators/BarriersPatients expressed mixed views about how much information should be provided. They said that information about adverse effects was complex and confusing. To support self‐monitoring of adverse effects, they desired information on what was normal and the clinical relevance of the tests, and thought that information would support shared decision making. They offered positive comments about the booklet content and format. However, they said that the booklet alone would not sufficiently empower patients, and that a one‐on‐one explanation of their role in decision making was needed.HarmsNR
Christensen^22^2004DenmarkReduce frequent attenders of after‐hours primary care serviceRandomized controlled trial	TheoryNRMultifacetedYesContentGeneral practitioners received information about the project and received 3 times the normal consultation fee; they were invited to an educational meeting about the treatment of frequent attenders. All general practitioners received a summary of discussions at educational meetings, and a contact list for included patients from their practice. Following an after‐hours contact, patients received an invitation to contact their general practitioner for a consultation.DeliveryNRTimingPhysicians received patient lists monthly. Patients were contacted 2‐5 d following an after‐hours contact.ParticipantsPatients who had 5 or more contacts during preceding 12 mo in 83 intervention and 93 control practices and physicians in those practicesPersonnelNR	OutcomesMain outcome measure was decrease in after‐hours contacts. Outcomes were reported for 3500 intervention and 4635 control patients from 83 intervention and 93 control practices. The number of contacts was fewer in the intervention group but significantly different only after 12 mo and for women aged 17‐66 y with 5‐9 contacts in the previous 12 mo. There were no significant differences between intervention and control patients for secondary outcomes (contacts with physicians, hospital admissions, visits to outpatient clinics). Only 44 (29%) of physicians attended 1 of 5 educational meetings, and 8.8% of patients participated in a consultation.Facilitators/BarriersNRHarmsNR
Scheel^23^2002NorwayRapid return to work after leave for low back painRandomized controlled trial	TheoryNRMultifacetedYesContentIntervention group #1 (passive strategy) included information targeted to patients and general practitioners; a new check box on the form for reporting sick leave intended to remind general practitioners to consider rapid return to work; standard agreement between employer and employee to facilitate a rehabilitation plan; and a desktop summary for general practitioners of clinical practice guidelines for low back pain emphasizing advice to stay active. Intervention #2 (proactive strategy) included the passive strategy plus an educational meeting for general practitioners on low back pain and rapid return to work; and a resource person for each region who followed up with patients to coordinate and communicate between patients, general practitioners and employers.DeliveryNRTimingNRParticipantsPatients absent from work for >16 d due to low back pain from all municipalities in 3 countiesPersonnelRegional resource people were physical therapists	OutcomesMain outcome measures were use of rapid return to work and length of sick leave. Outcomes were reported for 2232 patients in proactive intervention, 2045 patients in passive and 1902 patients in control group. Rapid return to work was used by significantly more patients in the proactive intervention group (17.7%) compared with the passive intervention group (10.8%) and the control group (12.4%) (χ^2^ = 5.67, *P* = .018). A significant difference was detected for the subgroup of patients who were on sick leave >4 wk (*P* = .016) but not the subgroup on sick leave for >12 wk (0.067).Facilitators/BarriersNRHarmsNR

NR, not reported.

### 3.3 Intervention characteristics

Table 2 summarizes the characteristics of interventions labelled as PMI in included studies. No studies explicitly defined PMI. Most often, the term “patient‐mediated” or “patient mediated” was employed in the article title, introduction or discussion. When referred to in Methods, little or no description was provided of what constituted a PMI, for example, “Clinics were informed about different patient‐mediated interventions…,”^17^ or “Patient‐mediated intervention:…patients received…an invitation to contact their [physician]….”^22^

**Table 2:** Characteristics of interventions labelled as patient‐mediated intervention (PMI)

Study	How PMI was described and defined by study authors	Intervention target	Intervention	Findings reported	Pathway (Figure 1)
McKellar^16^	DescriptionReferred to in Introduction: “Patient‐mediated interventions, in particular “Question‐Asking Tools” or “Prompt Sheets” have been documented as effective approaches to actively involve patients to implement informed and responsive self‐care.”Definition NR	Patients	Educational material (booklet, question prompt list) and education (2 visits with research coordinator) to support self‐management after stroke	Patient‐reported participation in valued activities, and patient feedback about educational material and its impact on question‐asking	1
Huppelschoten^17^	DescriptionReferred to in Methods: “Clinics were informed about different patient‐mediated interventions to enhance the communication with their patients (eg, organizing focus groups).”Definition NR	Patients	NR (intervention or its intent)Although not referred to as PMI, physicians received an audit and feedback report, educational outreach, information about PMIs they could implement, a newsletter, access to an online discussion group and follow‐up support from a researcher	Patient‐reported patient‐centredness of fertility care	2
Harris^18^	DescriptionReferred to in Title as “patient‐mediated practice change.”Definition NR	Patients	Educational material (evidence summaries, question prompt list) to support communication with physician about COPD	Patient feedback about educational material and its impact on question‐asking	1
Harris^19^	DescriptionReferred to in Introduction: “Patient mediated methods have potential in increasing doctors’ implementation of evidence.” and Discussion: “This intervention differed from previous patient mediated interventions by giving fuller evidence information and by covering a large number of treatments.”Definition NR	Patients	Educational material (evidence summaries, question prompt list) to support communication with physician about COPD	Patient‐reported influenza vaccination and bone density testing	1
Murie^20^	DescriptionReferred to in Title: “patients’ perceptions of patient‐mediated interventions,” Introduction: “a template for a ‘patient‐mediated intervention’,” Methods: “…participants then focused on describing what they considered to be an ‘ideal’ model of a PMI…” and Discussion: “…patients have contributed to the design of a PMI…”Definition NR	Patients	Educational material (booklet) to support self‐management of congestive heart failure	Patient feedback about educational material	1
Murie^21^	DescriptionReferred to in Title: “an evaluated patient‐mediated intervention” and Discussion: An evaluated patient‐mediated intervention for monitoring amiodarone…”Definition NR	Patients	Educational material (booklet) to support self‐monitoring of medication adverse effects	Patient feedback about educational material	1
Christensen^22^	DescriptionReferred to in Introduction: “Successful implementation change requires a combination of several intervention strategies that includes…patient‐mediated intervention” and Methods: “Patient‐mediated intervention: 2‐5 d after the index contact, patients received…an invitation to contact their GP for a status consultation.”Definition NR	Patients	Invitation to contact their physician for a consultation to address health‐care issues during regular work hoursAlthough not referred to as the PMI, physicians were invited to an educational meeting and received educational material to help them understand how to manage frequent attenders	Use of an after‐hours primary care service	1
Scheel^23^	DescriptionReferred to in Conclusions: “Relatively few trials have investigated the effects of patient‐mediated interventions on professional practice.”Definition NR	Patients (unclear what interventions were PMI)	Educational material (“information targeted to patients”) to support self‐management of low back painAlthough unclear if physician strategies were considered PMI, physicians received educational material, reminders and invitation to an educational meeting to help them understand how to support patient self‐management of low back pain	Return to work	1

NR, not reported.

In all studies, the intervention under development or evaluated was targeted to patients, although one study did not specify if interventions targeted to physicians were also considered part of the PMI strategy.^23^ The patient interventions implemented in one study were not reported.^17^ In other studies, the patient intervention was most frequently educational material (ie, booklets, question prompt lists, evidence summaries)^16^, ^18^, ^19^, ^20^, ^21^, ^23^ and, in one study, an invitation to contact their physician for an appointment.^22^

Three studies employed multifaceted strategies in which one or more components were targeted at physicians. In two of these studies, the physician interventions were not considered PMIs and consisted of audit and feedback, educational outreach and educational material in one study,^17^ and educational material and an educational meeting in the second study.^22^ In the third study that employed a physician‐targeted intervention, it was not specified if the physician intervention, comprised of educational material, invitation to an educational meeting and reminders, was considered as part of the PMI.^23^

### 3.4 Intervention impact

Details about intervention impact are summarized in Table 1. Of 5 studies that evaluated behavioural or clinical outcomes, there was no significant difference before and after intervention delivery or in comparison with control groups in 4 studies,^16^, ^17^, ^19^, ^22^ 2 of which included both patient and physician interventions although physician interventions were not considered PMIs.^17^, ^22^ In the fifth study, in which it was not clear if physician interventions were also considered part of the PMI, intervention patients with low back pain returned to work significantly earlier compared with control patients.^23^

Four studies assessed facilitators or barriers of PMIs through qualitative interviews or focus groups with patients. In 2 studies, patients said that an educational booklet alone was useful but interaction with health‐care professionals was also needed.^16^, ^21^ In another study of evidence summaries and question prompts, patients varied in views about their relevance and usefulness, and few said that it prompted them to ask questions.^18^ In another study of an educational booklet, patients said they appreciated information on self‐management and its positive tone, unambiguous guidance, visual appeal and small, portable size.^20^

### 3.5 Intervention pathways

Table 2 summarizes the intervention target, intervention and impact and, based on those details, categorizes the intervention addressed in each study according to PMI pathways depicted in Figure 1. For example, in one study, educational material was targeted at patients and its impact was evaluated based on patient‐reported behaviour.^16^ According to Figure 1, this corresponds to PMI pathway 1. Seven studies were based on PMI pathway 1 in which patient strategies are meant to influence patient behaviour leading to improved patient outcomes,^16^, ^18^, ^19^, ^20^, ^21^, ^22^, ^23^ although in one study, it was unclear whether physician interventions were also considered part of the PMI.^23^ One study was based on PMI pathway 2 in which patient strategies are meant to influence health‐care professional behaviour leading either directly to improved patient outcomes, or indirectly to improved patient outcomes by influencing patient behaviour.^17^ No studies explicitly involved interventions reflecting pathway 3 in which physician strategies are meant to influence patient behaviour leading to improved patient outcomes—although 3 studies included physician strategies, 2 did not refer to the physician interventions as PMI^17^, ^22^ and, in one, it was not clear.^23^ No studies were based on PMI pathway 4 in which physician strategies are meant to directly influence physician behaviour leading directly to improved patient outcomes, or indirectly to improved patient outcomes by influencing patient behaviour.

## 4 DISCUSSION

This scoping review described the characteristics of primary research on interventions that were explicitly labelled as “patient‐mediated” or “patient mediated” and compared the interventions to original conceptualizations of PMI that proposed at least 4 pathways (Figure 1).^9^, ^10^, ^11^ Following publications by Oxman^10^ and Davis^11^ in 1995 that identified PMIs, only a small number of primary studies published from 2002 to 2015 developed or evaluated interventions labelled as PMIs. None of the included studies defined PMIs or described what was meant by PMI, none of the PMIs developed or evaluated were based on theory, and 1 of 8 included studies demonstrated a significant improvement in patient behaviour.^23^ Although 3 of 8 studies employed physician interventions, only patient interventions, which were largely patient educational material, were considered PMIs. Therefore, 7 of 8 included studies were based on PMI pathway 1 in which patient interventions influence patient behaviour leading to improved patient outcomes. Overall, there has been little scientific advancement on PMIs—few primary studies explicitly investigated PMIs, and the conceptualization and operationalization of PMIs was unclear and limited to 1 of 4 possible pathways.

This review found that few studies investigated interventions explicitly labelled as PMI. Similarly, other reviews of behaviour change interventions also included few studies that were considered by the authors to be PMIs. For example, in a systematic overview published by Johnson et al in 2016 of 67 systematic reviews of the effectiveness of behaviour change interventions, 63 targeted providers only, 4 targeted both patients and providers, and none targeted patients alone.^3^ French et al reviewed studies of interventions to improve the appropriate use of imaging for people with musculoskeletal conditions, and of 28 included studies, 6 were considered to involve PMIs.^24^ Ostini et al. reviewed studies of interventions to improve prescribing, and of 29 included studies, 4 involved PMIs.^25^ Grimshaw et al^4^ reviewed studies of interventions to disseminate and implement guidelines, and of 235 included studies, 4 employed PMIs. A systematic review by Gagliardi et al of research published from 2005 to 2014 that evaluated interventions which informed, educated or activated patients with arthritis or cancer included only 16 studies and concluded that further research was needed to evaluate different types of PMIs for patients and providers.^26^

Patient‐mediated interventions can have moderate to large effects on health‐care delivery and associated patient outcomes either alone or in concert with health‐care professional interventions.^4^, ^5^ Therefore, the overall finding that little explicit PMI research has been undertaken since the concept emerged in 1995^10^, ^11^ reveals an urgent need for research in this area of implementation science. Specific scoping review findings suggest options for future research. For example, none of the included studies defined PMIs and, in most studies, only patient interventions that directly influenced patient behaviour (PMI pathway 1 or 2) were considered to be PMIs. No studies were identified that directly influenced health‐care professional behaviour (PMI pathway 3 or 4). This may be attributed to the fact that health‐care professional interventions, even those that provide health‐care professionals with patient data, which could be considered a form of PMI, are generally referred to using distinct labels such as audit and feedback that are consistently defined in checklists of behavioural interventions.^6^, ^7^ Intervention developers may not be fully aware of what constitutes a PMI, which is likely exacerbated by the lack of clarity in taxonomies of behaviour change interventions.^6^, ^7^ Given that this review found that only interventions targeted to patients were considered PMIs and interventions targeted to health‐care professionals are referred to using other standard labels, perhaps only those interventions directed at patients should be considered PMIs. International consensus among intervention developers is needed to consider the original conceptualizations of PMI^9^, ^10^, ^11^ as depicted by 4 PMI pathways in Figure 1 and clarify the definition of PMIs. It is unclear how systematic reviews of behaviour change interventions, more recent than those by Oxman and Davis,^10^, ^11^ that included interventions considered as PMIs but not necessarily labelled as such defined PMIs. Building on this scoping review, in ongoing research, a systematic overview of systematic reviews could identify if PMI is being more consistently defined by those who conduct research syntheses.

Studies included in this review provided no or little description of the PMIs developed or evaluated, and no PMIs were informed by explicitly named theory. In future evaluative studies, researchers should use criteria such as the WIDER reporting checklist.^15^ The WIDER checklist recommends describing: the intervention (approaches, strategies), change techniques used in the intervention and the causal processes targeted by the change techniques to achieve particular outcomes (theory, underlying mechanism), mode of delivery (intensity, duration, timing), intervention content (knowledge generated or shared), participants and their role (the characteristics of those sponsoring, delivering and receiving the intervention), setting and adherence or fidelity. Also, research is needed to identify, describe and test relevant theory by which to optimally design and/or evaluate PMIs.^27^

In all studies that described patient interventions, the intervention was educational material, which achieved positive impact in only one of those studies.^23^ Patient educational material is inconsistently effective, and many other types of interventions are available to inform, educate and activate patients.^28^, ^29^ Future research should establish which types of patient interventions are most effective in different contexts. For example, patient‐reported outcomes could serve as the basis for designing PMIs,^30^ and various types of communication and decision aids can improve patient outcomes.^31^, ^32^ Three studies also employed educational interventions targeted to physicians, although they were not explicitly considered part of the PMI,^17^, ^22^, ^23^ of which only one achieved a positive impact.^23^ A meta‐review of 25 systematic reviews that compared direct and indirect effect size and dose‐response of single and multifaceted strategies showed no benefit of multifaceted over single strategies.^33^ Yet other research showed that educational interventions aimed at both patients and providers may be more effective than targeting one group alone.^5^ Ongoing research should resolve this discrepancy regarding the number and type of PMI interventions needed to improve health‐care delivery and associated patient outcomes.

This study featured both strengths and limitations. In contrast to other reviews that included primary studies in which behavioural interventions were considered by review authors to be PMIs,^24^, ^25^ this is the first review to characterize interventions explicitly labelled in primary studies as PMIs as a means of commenting on the evolution of the underlying science. Robust scoping review methods were employed, including independent screening and data extraction. However, several issues may limit the interpretation and application of these findings. The relatively small number of included studies, in part reflecting the restriction of interventions being explicitly labelled as PMIs and thereby omitting the inclusion of interventions based on patient‐reported outcomes or communication tools, and the sparse detail in those studies provide limited insight on how PMIs can be operationalized. Although we searched multiple databases since their inception, we may not have identified all relevant studies. We did not search the grey literature, assuming that empirical research on PMIs would be found in indexed databases. As a scoping review, the findings describe the nature of research on PMIs and provide direction for future research, and hence, this review cannot suggest how to best operationalize PMIs or which PMIs to use in a given context. Future research, for example a recently registered Cochrane review protocol, will provide insight on the effectiveness of different types of PMIs that are not necessarily labelled as such.^12^

## 5 CONCLUSIONS

This scoping review found that few primary studies published subsequent to the emergence of the concept of PMIs in 1995^10^, ^11^ explicitly developed or evaluated PMIs, which were not defined or described, and largely restricted to 1 of 4 possible PMI pathways that focuses on patient interventions to change patient behaviour. Researchers should employ reporting criteria when publishing the findings of PMI research so that research users understand the nature of the PMI employed. Given patient centrality to health care and the demonstrated potential for PMIs to improve health‐care quality,^4^, ^5^ further research is needed to develop the PMI concept, establish the effectiveness of different types of PMIs and understand when and how to employ them, either alone or combined with other interventions. Ongoing research should establish consensus on an expanded definition and examples of PMIs and identify or develop and test relevant theory by which to design and/or evaluate PMIs.

## CONFLICT OF INTEREST

The authors declare that they have no conflict of interests.
